# Оптимальный уровень 25(OH) D в сыворотке крови в аспекте ассоциации с опорно-двигательными, метаболическими, неврологическими, аутоиммунными и инфекционными заболеваниями

**DOI:** 10.14341/probl13687

**Published:** 2026-01-18

**Authors:** Е. А. Трошина, Е. А. Пигарова, Т. Л. Каронова, Ф. Х. Дзгоева, В. Е. Радзинский, И. И. Баранов, О. М. Лесняк, Ю. Э. Доброхотова, И. В. Кузнецова, Н. В. Зароченцева, Г. Р. Байрамова, О. А. Радаева, Е. В. Екушева, Л. А. Суплотова, Е. В. Матушевская

**Affiliations:** Национальный медицинский центр эндокринологии им. академика И.И. ДедоваРоссия; Federal State Budgetary Institution «I.I. Dedov National Medical Research Center of Endocrinology» of the Russian Ministry of HealthRussian Federation; НМИЦ им. В.А. Алмазова Минздрава РоссииРоссия; Federal State Budgetary Institution «V.A. Almazov National Medical Research Center of Endocrinology» of the Russian Ministry of HealthRussian Federation; Российский университет дружбы народов им. Патриса ЛумумбыРоссия; Patrice Lumumba Peoples’ Friendship University of RussiaRussian Federation; ФГБУ «Национальный медицинский исследовательский центр акушерства, гинекологии и перинатологии им. академика В.И. Кулакова» Минздрава РоссииРоссия; Federal State Budgetary Institution «Academician V.I. Kulakov National Medical Research Center of Obstetrics, Gynecology, and Perinatology» of the Russian Ministry of HealthRussian Federation; ГБОУ ВПО «Северо-Западный государственный медицинский университет им. И.И. Мечникова» МЗ РФРоссия; State Budgetary Educational Institution of Higher Professional Education «North-Western State Medical University named after I.I. Mechnikov National Research Medical University of the Russian Ministry of HealthRussian Federation; ФГАОУ ВО «Российский национальный исследовательский медицинский университет им. Н.И. Пирогова» Минздрава Российской ФедерацииРоссия; Pirogov Russian National Research Medical University of the Russian Ministry of HealthRussian Federation; Международная ассоциация гинекологов, эндокринологов и терапевтов (МАГЭТ)Россия; International Association of Gynecologists, Endocrinologists, and Therapists (IAGET)Russian Federation; ГБУЗ МО МОНИКИ им. М.Ф. ВладимирскогоРоссия; Moscow Regional Research and Clinical Institute («MONIKI»)Russian Federation; ФГБОУ ВО МГУ им. Н.П. ОгареваРоссия; Federal State Budgetary Educational Institution of Higher Education, National Research Ogarev Mordovia State UniversityRussian Federation; Академия постдипломного образования ФГБУ «ФНКЦ ФМБА России»Россия; Academy of Postgraduate Education of the Federal State Budgetary Institution Federal Scientific and Clinical Center of the Federal Medical and Biological Agency of RussiaRussian Federation; ФГБОУ ВО Тюменский ГМУ Минздрава РоссииРоссия; Tyumen State Medical University of the Russian Ministry of HealthRussian Federation

**Keywords:** витамин D, дефицит витамина D, 25-гидроксивитамин D, метаболизм витамина D, колекальциферол, эргокальциферол, эндокринология, vitamin D, vitamin D Deficiency, 25-Hydroxyvitamin D, vitamin D metabolism, cholecalciferol, ergocalciferol, endocrinology

## Abstract

В данной статье представлен обзор современных исследований, посвященных определению целевых уровней витамина D в крови. В ней рассматриваются биохимические и метаболические свойства витамина D, а также сложности стандартизации измерений 25(OH)D и вариабельность пороговых значений в разных популяциях. В ней обсуждается неоднозначность научных данных и необходимость учета индивидуальных факторов при интерпретации уровня витамина D. Этот обзор уникален комплексным подходом к анализу влияния витамина D не только на здоровье костей, но и на иммунную и метаболическую функции, что расширяет постоянно развивающееся понимание клинической значимости витамина D. В данной работе подчеркивается важность персонализированных рекомендаций в назначении и дозировании витамина D, основанных на современных клинических данных и научных стандартах. Проведенный анализ подчеркивает необходимость персонализированного подхода к назначению препаратов витамина D для достижения и поддержания его уровня в крови от 30 до 60 нг/мл, при этом отмечается, что более высокие уровни могут потребоваться для людей с генетической или приобретенной резистентностью к нутриенту. Полученные данные обосновывают разработку основанных на фактических данных персонализированных клинических стратегий профилактики и лечения заболеваний, связанных с дефицитом витамина D. Синтезированные данные имеют важное значение для развития исследований и клинической практики в области эндокринологии, акушерства, дерматологии, неврологии и иммунологии.

## Abstract (en)

This article presents an overview of current research on determining target blood levels of vitamin D. It examines the biochemical and metabolic properties of vitamin D, as well as the challenges of standardizing 25(OH)D measurements and the variability of threshold values across populations. It discusses ambiguities in scientific data and the need to consider individual factors when interpreting vitamin D levels. This review is unique in its comprehensive approach to analyzing the effects of vitamin D not only on bone health but also on immune and metabolic functions, which broadens ever evolving understanding of the clinical significance of vitamin D. This work emphasizes the importance of personalized recommendations for vitamin D dosing and prescription based on current clinical data and scientific standards. Performed analysis highlights the need for personalized vitamin D supplementation to reach and maintain blood levels between 30 and 60 ng/mL, noting that higher levels might be necessary for people with genetic or acquired resistance. These insights substantiate the development of evidence-based, personalized clinical strategies for the prevention and treatment of vitamin D deficiency-related disorders. The synthesized data offer significant implications for advancing research and clinical practice in endocrinology, obstetrics, dermatology, neurology and immunology.

## ВВЕДЕНИЕ

Дефицит витамина D является одним из наиболее распространенных в мире дефицитов нутриентов, затрагивающим, по данным эпидемиологических исследований, около 1 млрд человек [1]. Известно, что основной биологической ролью витамина D в организме человека является регуляция метаболизма кальция и фосфора. Так, витамин D оказывает положительное влияние на всасывание кальция в кишечнике, увеличивает реабсорбцию кальция в почках, а также поддерживает минерализацию костей путем регуляции дифференцировки хондроцитов и остеобластов [2].

Результаты проведенных исследований продемонстрировали связь низкого уровня обеспеченности витамином D с повышенным риском развития различных социально-значимых заболеваний, включая заболевания опорно-двигательного аппарата, метаболические, сердечно-сосудистые, злокачественные и инфекционные заболевания. Также установлен вклад дефицита витамина D в патогенез неврологических, аутоиммунных и эндокринных заболеваний [3].

Учитывая высокую распространенность дефицита витамина D, исследований, демонстрирующих плейотропные и мультимодальные эффекты витамина D в зависимости от уровня 25(ОН)D в крови, по-прежнему крайне мало. Это, вероятно, связано с тем фактом, что любые иные эффекты витамина D, не связанные с фосфорно-кальциевым обменом, стали объектом внимания исследователей относительно недавно, и большинство работ сосредоточены на изучении скелетных эффектов витамина D.

Учитывая высокую распространенность дефицита витамина D, наличие экспериментальных исследований, посвященных плейотропным и мультимодальным эффектам витамина D, гипотезы по применению этого витамина-прогормона для профилактики целого ряда социально-значимых заболеваний активно обсуждаются во всем мире. В рамках этого экспертного совета выполнен анализ и обобщен материал по существующим экспериментальным и клиническим данным, касательно эффектов витамина D на различные органы и системы [4][5] и предложены возможные пути реализации классических и плейотропных эффектов витамина D для здоровья населения.

## ОПРЕДЕЛЕНИЕ И ЭПИДЕМИОЛОГИЯ

Дефицит витамина D — это состояние, характеризующееся снижением концентрации 25(ОН)D в сыворотке крови ниже оптимальных значений, которое может потенциально приводить к снижению всасывания кальция в кишечнике, развитию вторичного гиперпаратиреоза и повышению риска переломов, особенно у пожилых лиц [2].

Дефицит и недостаточность витамина D определяется как уровень 25(OH)D в сыворотке крови менее 20 нг/мл и от 20 до 30 нг/мл соответственно [2]. Анализ 14 популяционных исследований, оценивающий распространенность дефицита витамина D в странах Европы, продемонстрировал, что среди 55 844 европейских жителей различного возраста уровень 25(ОН)D крови ниже 12 нг/мл наблюдался у 13% обследованных, с выраженными сезонными отличиями (доля уровня 25(ОН)D ниже 12 нг/мл составила 18% в период с октября по март и 8% с апреля по ноябрь). Уровень ниже 20 нг/мл был выявлен у 40% обследованных [6][7].

Результаты, полученные в рамках исследования распространенности дефицита и недостаточности витамина D в Российской Федерации, согласуются с мировыми данными в отношении сезонных колебаний, но значительно превосходят по своему масштабу значения распространенности дефицита и недостаточности витамина D в других странах во многом из-за географического расположения Российской Федерации, а также отсутствия централизованного обогащения продуктов питания данным нутриентом. Так, уровень 25(ОН)D крови менее 30 нг/мл диагностирован у 70–95% обследованных лиц взрослой популяции [8–14].

Следует отметить, что первое многоцентровое регистровое исследование, проведенное в Российской Федерации, продемонстрировало наличие уровня 25(ОН)D крови ниже 20 нг/мл у 56% обследованных лиц в весенний период (март-май) и у 26% — в осенний период (октябрь-ноябрь) наблюдения, а уровня ниже 30 нг/мл — у 84% и 62% соответственно [15]. Приведенные данные согласуются с результатами когортного проспективного исследования по оценке уровня 25(ОН)D у беременных женщин, продемонстрировавшими наличие низкого уровня витамина D в I триместре беременности в 84,3% случаев независимо от времени года проведения обследования [16]. Схожие данные по распространенности дефицита данного нутриента в Российской Федерации были представлены и в начале пандемии COVID-19 [17].

## МЕТАБОЛИЗМ ВИТАМИНА D

Известно, что витамин D поступает в организм двумя путями: посредством синтеза колекальциферола (D3) из предшественника 7-дегидрохолестерина в коже под воздействием ультрафиолетового (УФ) излучения В типа, а также с пищей в виде витамина D животного происхождения — холекальциферола (D3, МНН лекарственного препарата — колекальциферол) и растительного происхождения — эргокальциферола (D2).

Этапы метаболизма для обеих форм витамина D (D2 и D3) являются общими и включают первый этап гидроксилирования ферментами CYP2R1 и CYP27A1 в печени с образованием кальцидиола (25(OH)D) и второй этап — при помощи фермента CYP27B1, главным образом в почках, до активного метаболита кальцитриола — 1,25(OH)2D. Основная функция 1,25(OH)2D заключается в поддержании гомеостаза кальция и фосфора. Однако связываясь с внутриклеточными специфическими рецепторами витамина D (VDR) в тканях, 1,25(OH)2D запускает множество метаболических внекостных процессов.

В отличие от почечной CYP27B1, экстраренальные формы фермента, которые опосредуют многочисленные плейотропные эффекты, регулируются не посредством передачи сигналов от паратиреоидного гормона (ПТГ), фактора роста фибробластов (FGF-23), кальция или фосфатов, а с помощью регуляторных факторов, зависящих от конкретной функции. Следует также отметить, что регуляция экстраренальной CYP27B1 зависима от концентрации циркулирующего 25(OH)D в крови [3][18][19].

## ПЛЕЙОТРОПНЫЕ ЭФФЕКТЫ ВИТАМИНА D

Результаты ранее проведенных исследований продемонстрировали, что витамин D регулирует клеточный цикл и, таким образом, оказывает существенное влияние на функционирование органов и систем человека. Связываясь c рецепторами VDR на клетках иммунной, нервной, пищеварительной, репродуктивной и сердечно-сосудистой систем, витамин D оказывает регулирующее, противовоспалительное, антипролиферативное, а также антифибротическое действия [20].

В литературе имеются немногочисленные сведения об оптимальных пороговых значениях концентрации 25(OH)D в сыворотке крови для реализации плейотропных эффектов, которые варьируют от 25 нг/мл до 60 нг/мл [21–23]. Эти концентрации 25(ОН)D согласуются с рекомендациями Российской ассоциации эндокринологов, где целевые уровни прописаны как 30–60 нг/мл [2].

Необходимо признать, что в отличие от воздействия витамина D на обмен кальция, его внекостные плейотропные эффекты намного сложнее оценить в клинической практике. На основании когортных исследований была выдвинута гипотеза о том, что для реализации плейотропных эффектов нужны более высокие уровни 25(ОН)D в сыворотке крови. Так, например, были выявлены ассоциации меньшей частоты онкологических, кардиоваскулярных, аутоиммунных заболеваний, развития сахарного диабета (СД), падений, переломов даже летальности с более высокими значениями 25(ОН)D в сыворотке крови [24][25]. Вместе с тем, все эти различия были получены на основании анализа клинических исходов в больших когортах населения. Однако в подобных исследованиях может быть сложно доказать причинно-следственную связь между одним фактором, например, уровнем обеспеченности витамином D и клиническим исходом, в частности — развитием онкологических или иных заболеваний.

Для понимания дополнительной пользы, ассоциированной именно с приемом витамина D, были проведены широкомасштабные рандомизированные контролируемые исследования с оценкой эффективности при достижении уровня 25(ОН)D 40 нг/мл и 50 нг/мл, и конечными точками в виде летальности, развития онкологических заболеваний, сахарного диабета 2 типа (СД2), падений, переломов, а также кардиоваскулярных событий. В упомянутых исследованиях не удалось получить существенных различий по основным исходам для групп пациентов, принимавших препараты витамина D [26–31]. Все эти исследования полностью соответствовали критериям проведения рандомизированных клинических исследований медицинских препаратов. Однако имелся ряд ограничений: популяции, включенные в большинство протоколов, не были ограничены только пациентами с исходным дефицитом витамина D; в некоторых из них, в группах плацебо, допускался прием профилактических доз колекальциферола (≤800 МЕ в сутки); в некоторых работах не учитывались индивидуальные особенности метаболизма витамина D у включенных пациентов [32].

Таким образом, принимая во внимание результаты проведенных клинических, эпидемиологических и когортных исследований, можно говорить о потенциальной пользе приема витамина D с целью улучшения показателей здоровья и качества жизни, в частности для снижения риска СД2, острых респираторных вирусных инфекций, влияния на репродуктивные исходы и т.д. Следует подчеркнуть, что потребность в дозах витамина D и «оптимальный уровень 25(ОН)D» могут различаться в зависимости от поставленных целей (табл. 1). Проведение дальнейших крупных исследований требует персонализированного подхода с акцентом на оценку эффектов терапии витамином D в группах с различной степенью выраженности дефицита.

**Table:** Таблица 1. Плейотропные эффекты витамина D и связанные с ними механизмы и нозологии Примечание: ХОБЛ — хроническая обструктивная болезнь легких; ГнРГ — гонадотропин-рилизинг-гормон; ЛГ — лютеинизирующий гормон; ФСГ — фолликулостимулирующий гормон; СПКЯ — синдром поликистозных яичников; ФНО-α — Фактор некроза опухоли-альфа.

Влияние на различные системы организма	Механизм развития и клинические эффекты	Предполагаемый оптимальный уровень 25(OH)D (нг/мл)
Опорно-двигательная система [33]	1,25(OH)2D взаимодействует с ядерным рецептором витамина D (VDR) в тонком кишечнике, увеличивая экспрессию эпителиального кальциевого канала и связывающего кальций белка, что приводит к повышению усвоения кальция из рациона. Позитивный клинический эффект — эффективное всасывание кальция в кишечнике, благоприятный ответ на терапию бисфосфонатами	>30
Клетки и ткани иммунной системы [22][33–38]	Влияние на дифференцировку активных CD4+ Т-клеток; усиление ингибирующей функции Т-клеток; дифференцировка моноцитов в макрофаги с повышением антибактериальной и противовирусной активности; подавление IL-12, γ-интерферона, иммунных ответов Th1; подавление TGF-β/Smad3. Позитивный клинический эффект — снижение частоты рецидивирующих инфекций, респираторных заболеваний (грипп, туберкулез, ХОБЛ), синдрома хронической усталости, болезни Бехчета, воспалительных заболеваний кишечника, ревматоидного артрита	>50
Антипролиферативное, антифибротическое действие [37][39–42]	Индукция апоптоза в злокачественных клетках (взаимодействие Bcl2/Bax); влияние на нейротрофические факторы; ингибирование цикла роста TGF-α/EGFR, снижение пролиферации кератиноцитов. Позитивный клинический эффект — снижение частоты представленности/заболеваемости рака простаты, молочной железы, толстой кишки, миелопролиферативных заболеваний и смертности от всех причин	≥40
Сердечно-сосудистая система [37][43–45]	Обратная связь с ренин-ангиотензиновой системой, регуляция артериального давления и электролитного баланса; регуляция процесса гипертрофии клеток миокарда; подавление воспалительных цитокинов, ангиогенеза и кальцификации сосудов. Позитивный клинический эффект — снижение представленности и выраженности артериальной гипертензии, инфаркта миокарда, застойной сердечной недостаточности, атеросклероза	≥40
Нервная система [46–54]	Нейротрофическое (влияние на фактор роста нервов NGF), нейропротективное (влияние на процессы синаптической пластичности), противовоспалительное, антиоксидантное (подавление окислительного стресса в нейронах и микроглии), антиноцицептивное действие, регуляция дофаминовой и серотониновой передачи; оптимальное функционирование нейронов коры головного мозга. Позитивный клинический эффект — улучшение когнитивного функционирования; снижение риска развития нейродегенеративных, аутоиммунных неврологических заболеваний, нейропсихологических расстройств; нарушений сна; уменьшение частоты и интенсивности первичной головной боли (мигрени)	>40
Репродуктивная система [55–57]	Регуляция экспрессии генов, участвующих в синтезе и метаболизме эстрогена; повышение выработки ароматазы; регуляция высвобождения ГнРГ, ЛГ, ФСГ; усиление функции желтого тела и прогестерона. Позитивный клинический эффект — нормализация менструального цикла, снижение представленности и выраженности СПКЯ, гестационного сахарного диабета, преэклампсии и преждевременных родов	>40
β-клетки поджелудочной железы [58]	Защита и предотвращение разрушения β-клеток, снижение аутоиммунного поражения за счет ингибирования провоспалительных цитокинов (ФНО-α). Позитивный клинический эффект — снижение риска более раннего возникновения и более тяжелого течения сахарного диабета 1 и 2 типов	>50
Резистентность к витамину D [36– 38]	Генетически обусловленная или приобретенная резистентность к витамину D является ключевым патогенетическим механизмом аутоиммунных заболеваний, включая псориаз. Витамин D регулирует функцию кератиноцитов, ключевых клеток в патогенезе псориаза. Позитивный клинический эффект — снижение представленности и выраженности аутоиммунных заболеваний, рассеянного склероза, псориаза	≥80

## КОРРЕКЦИЯ СТАТУСА ВИТАМИНА D

Диапазон концентрации 25(OH)D 30–40 нг/мл обычно может быть достигнут приемом витамина D в дозах 2000–4000 МЕ/день. Для достижения значений выше 40 нг/мл в большинстве случаев требуются более высокие дозы колекальциферола [59].

Результаты исследований продемонстрировали, что под воздействием на все тело одной минимальной эритематозной дозы имитируемого солнечного света в коже может вырабатываться от 10 000 до 25 000 МЕ витамина D [60]. Таким образом, логично предположить, что такие дозы витамина D можно считать безопасными. Подтверждением этому факту могут служить данные опубликованных работ, в которых сообщалось о безопасности приема высоких доз витамина D. В частности, результаты наблюдения за 3882 участниками, включенными в исследование в Канаде в период с 2013 по 2015 гг., находятся в открытом доступе и свидетельствуют об эффектах приема витамина D3 в дозе до 15 000 МЕ/день в течение 6–18 месяцев. Целью данного исследования было определение доз витамина D, необходимых для достижения концентрации 25(OH)D>40 нг/мл. Было обнаружено, что для достижения целевой концентрации 25(ОН)D участники с нормальным ИМТ должны принимать не менее 6000 МЕ витамина D в день, тогда как участники с избыточным весом и ожирением — 7000 МЕ/день и 8000 МЕ/день соответственно. Важно отметить тот факт, что достижение в редких случаях концентрации 25(OH)D в сыворотке крови до 120 нг/мл не были ассоциированы ни с нарушением гомеостаза кальция, ни с проявлением токсичности [61].

Интересны также результаты другого исследования, в котором 777 длительно госпитализированных пациентов, принимали от 5000 до 50 000 МЕ/день витамина D. В ходе наблюдения было установлено, что подгруппа больных, принимавших витамин D в дозе 5000 МЕ/день, достигла средних концентраций 25(OH)D 65±20 нг/мл через 12 месяцев терапии, тогда как пациенты, принимавшие 10 000 МЕ/день, достигли уровня 25(OH)D 100±20 нг/мл. Необходимо отметить, что ни у одного из пациентов, достигших концентрации 25(OH)D в диапазоне 40–155 нг/мл, не развилась гиперкальциемия, нефролитиаз или какие-либо другие симптомы, характерные для клинических проявлений передозировки витамина D [62].

Необходимо также остановиться на результатах единственного, проведенного на территории Российской Федерации открытого многоцентрового сравнительного рандомизированного клинического исследования III фазы, целью которого была оценка эффективности и безопасности терапии препаратом Фортедетрим, в сравнении с терапией препаратом Вигантол®, у пациентов с дефицитом витамина D [63]. В исследовании приняли участие 150 пациентов, рандомизированных на 3 группы (по 50 человек в каждой), в которых для насыщающих доз в группах 1(Т) и 2(R) использовали капсулы Фортедетрим соответственно 50 000 МЕ 1 раз в неделю (5 капсул по 10 000 МЕ) и 8000 МЕ ежедневно (2 капсулы по 4000 МЕ), а в группе сравнения 3(Х) — Вигантол® по 1000 МЕ ежедневно (2 капли). Сравнительные результаты лечения наглядно продемонстрированы на графике (рис. 1).

**Figure:**
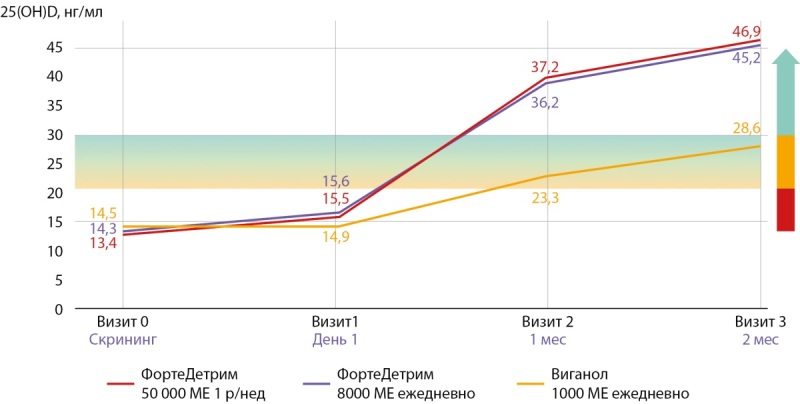
Рисунок 1. Изменение концентрации 25(ОН)D в сыворотке крови после 2-месячного лечения дефицита витамина D [63].

Как видно из представленных на графике данных, терапия препаратом Фортедетрим в дозах 8000 МЕ ежедневно или 50 000 МЕ 1 раз в неделю в 1-й и 2-й группах соответственно, по сравнению с группой сравнения, принимавшей витамин D в дозе 1000 МЕ, не только приводила к быстрому повышению уровня 25(ОН)D в сыворотке крови через месяц терапии, но и не ассоциировалась с различием в количестве и выраженности нежелательных и побочных явлений [63].

Наконец, хотелось бы отметить, что на сегодняшний день актуальной для здравоохранения остается необходимость устранения дефицита витамина D в популяции, с достижением концентрации 25(OH)D в сыворотке крови не ниже 20 нг/мл. Для этого, помимо здорового образа жизни, включающего правильное питание и физическую активность, необходим прием адекватных доз препаратов витамина D. Алгоритм назначения препаратов витамина D в зависимости от исходного уровня 25(OH)D представлен на рисунке 2.

**Figure:**
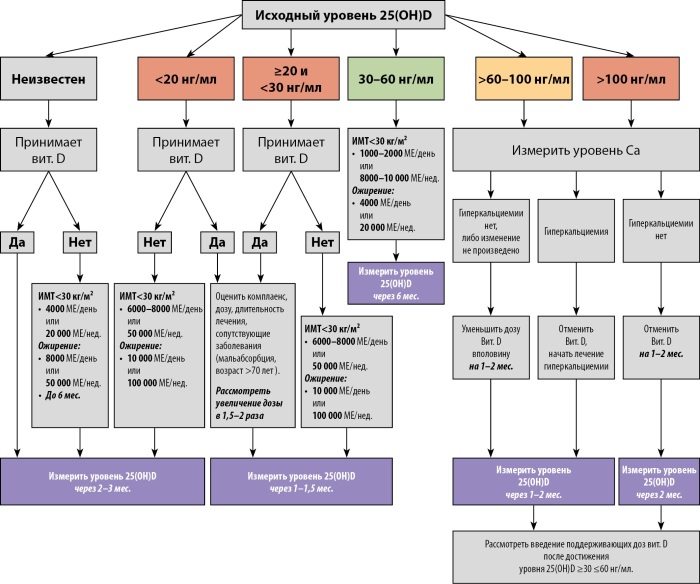
Рисунок 2. Алгоритм назначения препаратов витамина D для достижения оптимальных значений 25(ОН)D в сыворотке крови [2][64].

## ЗАКЛЮЧЕНИЕ

Учитывая многочисленные данные о значительной пользе достижения уровня 25(ОН)D в сыворотке крови выше 30–40 нг/мл для здоровья и отсутствии побочных эффектов при этом, целесообразно подходить к применению препаратов витамина D с должной ответственностью. Установление индивидуальных пороговых значений 25(ОН)D для плейотропных и мультимодальных эффектов витамина D должно учитывать нозологию, возраст, вес, пол и этническую принадлежность пациентов.

Оптимальными значениями 25(ОН)D в сыворотке крови считается диапазон от ≥30 нг/мл до ≤60 нг/мл, однако для лиц с генетически обусловленной или приобретенной резистентностью к витамину D, возможно, потребуется превышение верхней границы. Также необходимо учитывать, что пациентам с определенными патологическими состояниями, такими как ожирение и сахарный диабет, а также принимающим препараты, влияющие на метаболизм витамина D, могут потребоваться дозы, превышающие общепопуляционные лечебные, поддерживающие и профилактические.

Таким образом, определение методов достижения и поддержания оптимальных уровней витамина D в крови должно базироваться на индивидуальном подходе с учетом комплекса факторов и клинических данных. Эти выводы имеют важное практическое значение для эндокринологии и смежных дисциплин, способствуют развитию персонализированной медицины и обеспечивают повышение эффективности профилактики и лечения заболеваний, связанных с дефицитом витамина D.

## ДОПОЛНИТЕЛЬНАЯ ИНФОРМАЦИЯ

Источники финансирования. Работа выполнена при поддержке компании «Акрихин».

Конфликт интересов. Авторы декларируют отсутствие явных и потенциальных конфликтов интересов, связанных с содержанием настоящей статьи.

Участие авторов. Все авторы одобрили финальную версию статьи перед публикацией, выразили согласие нести ответственность за все аспекты работы, подразумевающую надлежащее изучение и решение вопросов, связанных с точностью или добросовестностью любой части работы.
